# Clinical, social, and psycho-oncological needs of adolescents and young adults (AYA) versus older patients following hematopoietic stem cell transplantation

**DOI:** 10.1007/s00432-020-03419-z

**Published:** 2020-10-14

**Authors:** Kristin Pulewka, Bernhard Strauss, Andreas Hochhaus, Inken Hilgendorf

**Affiliations:** 1 Institut für Psychosoziale Medizin, Psychotherapie und Psychoonkologie, Universitätsklinikum Jena, Jena, Germany; 2 Klinik für Innere Medizin II, Abteilung Hämatologie und Internistische Onkologie, Universitätsklinikum Jena, Am Klinikum 1, 07740 Jena, Germany

**Keywords:** Stem cell transplantation, Quality of life, Psycho-oncology, Informational need

## Abstract

**Purpose:**

To analyze demand for information and advice as well as medical, psychological, and social needs of adolescents and young adults (AYAs) and older patients (non-AYA) after hematopoietic stem cell transplantation (HSCT).

**Methods:**

A questionnaire was sent to 100 HSCT recipients comprising *n* = 50 AYAs (aged 19–39 years) and *n* = 50 non-AYAs (> 39 years). The questionnaire covered the categories: (a) patient characteristics; (b) need for advice, on medical, psychological, and social care topics; (c) medical, psychological, and social needs, and (d) preferred forms and channels of information.

**Results:**

The return rate was 65%. 62.5% of patients indicated medical needs; 41.1% psychological needs, and 64.9% had needs concerning social issues. Among medical aspects, aftercare was important to both groups. Nutrition was of highest interest for AYA, while non-AYAs identified fatigue and vaccination as their most pressing concerns. Body shape/sexuality and relaxation techniques were the most common psychological issues for AYA, while coping strategies were important for both cohorts. Family, relationship and friends were of less interest in both groups. Rehabilitation and premature retirement were of highest interest for both cohorts. The preferred mode of communicating advice was one-to-one conversation in a quiet environment as opposed to informational sessions.

**Conclusion:**

Despite well-established aftercare programs following HSCT, many patients describe unmet needs regarding medical, psychological, and social policy issues. AYA and non-AYA differ in informational needs after HSCT, and, therefore, age-appropriate informational materials are necessary. Particularly AYA may profit from information covering body-shape/sexuality and nutrition, while both cohorts require information covering coping strategies and aftercare.

## Introduction

Hematopoietic stem cell transplantation (HSCT) is a potentially curative and intensive therapy for patients suffering from malignant hematologic diseases. Advances in the management of acute and delayed adverse events have increased patients’ life expectancy and quality of life considerably. However, after surviving the initial event, HSCT patients often are faced with burdens such as readmissions, slow recovery, long-term complications, uncertainty regarding lifestyle and fears about the future (Herzberg et al. 2013; Reinfjell et al. 2017). Although recommendations regarding medical survivorship issues after HSCT are available (Buchsel 2009; Tichelli et al. 2012; Hilgendorf et al. 2015; Tichelli and Rovó 2015), questions regarding physical functioning (van Haren et al. 2013; Persoon et al. 2013) and lifestyle have been insufficiently evaluated to date (Adelstein et al. 2014).

Within the population of cancer patients, adolescent and young adults (AYA) comprise a unique group requiring special care and attention. Nearly all elements of personal life are in a phase of re-organisation and instability (e.g. starting a career, emancipating from parents etc.). In addition, risk behavior and changes in local or personal bonds are common (Thomas et al. 2010; Treadgold and Kuperberg 2010). While challenges for AYA survivors who underwent HSCT in childhood have been reported (Cupit et al. 2016; Lahaye et al. 2017; Tremolada et al. 2018), data are sparse on AYA diagnosed and transplanted between the ages of 18 and 39 years. As the disease occurs in early life, AYA may experience fears of long-term effects. The HSCT experience can take them out of the mainstream of a dynamic life (Cooke et al. 2011), resulting in special informational needs on topics such as education and employment, social relationship and functioning, psychological issues and end-of-life challenges (Husson et al. 2018). To enable a return to daily life as soon as possible, the specific informational requirements of AYA must be addressed, as they may differ from the needs of older adult patients.

All HSCT recipients at our clinic receive a patient brochure with extensive information on the transplantation process, behavior recommendations for the posttreatment phase as well as contact persons for specific questions (e.g. for psychological or social care issues). Psychological care during in- and outpatient treatment is offered to all patients on demand and social policy aspects can be discussed on patients request with a social service worker during inpatient treatment. Before discharge after HSCT each patient receives a survivorship care plan with included vaccination recommendations and a personal medical consultation with detailed information.

Besides their survivorship follow-up care plan, HSCT recipients may have unmet medical and psychosocial needs. Therefore, the aim of this study is to expand upon previous research by examining unmet informational needs among those with HSCT and to evaluate whether these needs differ between AYA and non-AYA patients.

## Materials and methods

### Data collection

With an expected transplantation rate between 5 to 10 AYA per year at the Jena University Hospital, an estimated sample size of *n* = 50 AYA (defined as aged between 19 and 39 years at the time of HSCT) were anticipated for this study within the observation period between 2010 and 2017. A questionnaire was sent to 100 patients comprising these *n* = 50 AYA and *n* = 50 non-AYA (defined as aged above 39 years at the time of HSCT). Patients were eligible for study inclusion if they were over 18 years old and received HSCT at our institution. Patients were excluded from study participation if they did not provide written consent, they were cognitively not able to participate in the study or unable to speak German language. There was a letter explaining the aims of the study and a stamped addressed return envelope. All letters were delivered by mail, and no letters were returned because of an invalid address. Patients were contacted only once, and there was no reminder letter sent to the patient cohort.

### Questionnaire

 To expand and optimize our information offer, we created a questionnaire evaluating the need for advice after HSCT. The questionnaire was developed by medical, psychological and nursing staff at our institution based on discussion of collected patient requests and the team´s multidisciplinary expertise.

The questionnaire consisted of 33 items in four principal categories: (1) demographics, (2) general informational requirements, (3) need for advice on medical and psychosocial aspects, and (4) the preferred channel of information.

Answers regarding informational needs were rated on 6-point Likert scale ranging from 1—very low to 6—most intensive informational need. Questions arising in the course of daily care were collected and assigned to the four categories mentioned above. There was no preceding validation process before the implementation of this study. Therefore, the evaluation may be interpreted in terms of a pilot study. The questionnaire itself is included in File S1 of the supplemental material. The questionnaire was filled out and returned anonymously to the hospital.

### Statistical analyses

Data analysis was performed on all returned questionnaires. Results related to categorical data are reported as absolute frequencies. Group comparisons were performed using the Mann–Whitney *U* test. Patients who underwent autologous transplantation were excluded from GvHD ratings. Questionnaire ratings are reported descriptively using the median and 95% confidence intervals or interquartile range (IQR). Calculations were performed using IBM SPSS Statistics Version 21.

### Ethics

The study was approved by the institutional Ethics Committee (4317-01/15). All patients provided written informed consent on separate forms when returning the questionnaire. To guarantee the anonymous character of the study questionnaires and informed consent were separated.

## Results

### Patient characteristics

Sixty-five of the 100 (65%) questionnaires were returned and eligible for evaluation. Participating patients had a median age of 42 years (median: 42, range 21–63 years). The majority were of male gender (*n* = 40 (61.5%)). AYA (*n* = 30) had a median age of 32 (range 21–39) years and non-AYA (n = 35) of 55 (range 40–63) years. Gender distribution was similar in both groups (Table 1). The majority of AYA was unmarried (*n* = 18 (60%)), while 27 (77.1%) of the non-AYA reported to be married or in a close relationship, p = 0.001 (*χ*^2^ test). Whereas 33.3% (*n* = 10) of the AYA reported having children, 82.9% (*n* = 29) of non-AYA were parents, *p* = 0.001 (*χ*^2^ test). The most common reason for HSCT in our cohort was acute myelogenous leukemia, followed by multiple myeloma and acute lymphoblastic leukemia. Most of the patients underwent allogeneic HSCT (*n* = 39 (60.0%)). The median time from transplantation to survey was 3 years (IQR 2.0–4.9). Table 1 shows all patient characteristics.

**Table 1:** Patient characteristics of the total cohort and AYA/non-AYA subgroups

	AYA (*n* = 30)	Non-AYA (*n* = 35)	Total cohort (*n* = 65)
Median age [25th–75th percentile]	32 [29.0–36.3]	55 [50.0–59.0]	42 [33–56.5]
Male gender, *n* (%)	17 (56.7)	23 (65.7)	40 (61.5)
Graduation, *n* (%)			
No graduation	1 (3.3)	0 (0.0)	1 (1.5)
Lower secondary education	6 (20)	11 (31.4)	17 (26.2)
Secondary school level	11 (36.7)	20 (57.1)	31 (47.7)
High School	9 (30)	2 (5.7)	11 (16.9
University degree	6 (20)	5 (14.3)	11 (16.9)
Family status, *n* (%)			
Married/close relationship	11 (36.7)	27 (77.1)	38 (58.5)
Unmarried	18 (60.0)	3 (8.6)	21 (32.3)
Divorced	1 (3.3)	4 (11.4)	5 (7.7)
Widowed	0 (0.0)	1 (2.9)	1 (1.5)
Children, *n* (%)	10 (33.3)	29 (82.9)	39 (60.0)
Underlying diseases, *n* (%)			
Acute myelogenous leukemia	8 (26.7)	8 (22.9)	16 (24.6)
Multiple myeloma	2 (6.7)	11 (31.4)	13 (20.0)
Acute lymphoblastic leukemia	5 (16.7)	3 (8.6)	8 (12.4)
Non-Hodgkin's lymphoma	4 (13.3)	5 (14.3)	9 (13.8)
Myelodysplastic syndrome	2 (6.7)	4 (11.4)	6 (9.3)
Hodgkin's lymphoma	5 (16.7)	0 (0.0)	5 (7.7)
Aplastic anemia	2 (6.6)	1 (2.9)	3 (4.6)
Chronic myeloid leukemia	1 (3.3)	1 (2.9)	2 (3.1)
Solid tumor	1 (3.3)	0 (0.0)	1 (1.5)
Chronic lymphocytic leukemia	0 (0.0)	1 (2.9)	1 (1.5)
Primary myelofibrosis	0 (0.0)	1 (2.9)	1 (1.5)
Mode of transplantation, *n* (%)			
Allogeneic	19 (63.3)	20 (57.1)	39 (60.0)
Autologous	11 (36.7)	15 (42.9)	26 (40.0)
Time since transplantation, *n* (%)			
1 year	4 (13.3)	0 (0.0)	4 (6.2)
2 years	8 (26.7)	7 (20.0)	15 (23.1)
3 years	4 (13.3)	23 (65.7)	27 (41.5)
4 years	4 (13.3)	3 (8.6)	7 (10.8)
5 years	6 (20.0)	0 (0.0)	6 (9.2)
6 years	1 (3.3)	1 (2.9)	2 (3.1)
7 years	3 (10.0)	1 (2.9)	4 (6.2)

### General informational needs

Asked about their unmet needs for information on disease-related issues, 62.5% (*n* = 35) of patients identified requirements on medical, 41.1% (*n* = 23) on psychological and 64.9% (*n* = 37) on social care issues. Although not significantly different, more than the half of AYA patients reported psychological needs (51.9% (*n* = 14) vs. 31.0% (*n* = 9) of non-AYA, *p* = 0.114 (*χ*^2^ test)). With respect to the type of transplantation (autologous versus allogeneic HSCT), AYA patients receiving allogeneic HSCT revealed more requirements of psychological issues compared to non-AYA patients, *p* = 0.037 (*χ*^2^ test). Time since transplantation (< 3 years vs. > 3 years) had no impact on the general need for information.

### Medical informational needs

All participants were asked to rate their personal needs for advice, ranging from very low to most intensive. In terms of medical issues, the topics of aftercare (median grade 5 [95% CI 3.97–4.81]) and vaccination (median grade 5 [95% CI 4.12–4.97]) were most important. These were followed by nutrition (median grade 4 [95% CI 3.35–4.30]), medication (median grade 4 [95% CI 3.28–4.17]), physical activity and exercise (median grade 4 [95% CI 3.25–4.16]), GvHD (median grade 4 [95% CI 3.09–4.17]) and alternative medicine (median grade 4 [95% CI 3.17–4.20]). Aspects regarding skin / alopecia (median grade 3 [95% CI 2.84–3.67]) were of relatively minor importance.

**Fig. 1:**
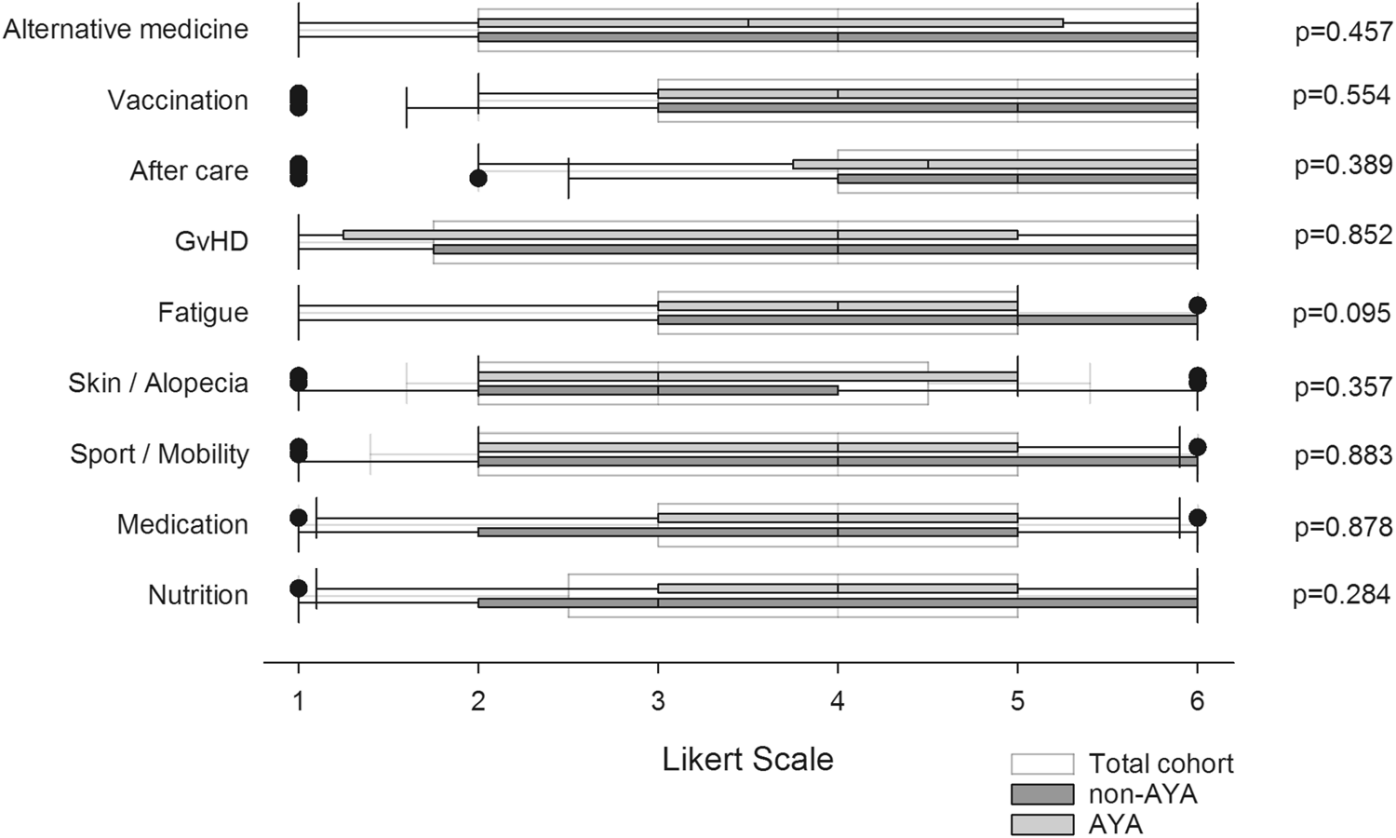
Informational needs regarding medical issues. Boxplots represent median values on a one-to-six graded Likert scale. White boxplots represent the total cohort, grey plots the subgroup of adolescents and young adults (AYA) and dark grey plots represent Adults > 39 years of age (non-AYA). Group comparisons were performed using non-parametric *U* test by Mann and Whitney. The referring *p* values are reported. *GvHD* graft versus host disease

In comparing AYA and non-AYA patients, AYA rated nutrition one point higher on the Likert scale than non-AYA (median grade 4 [95% CI 3.45–4.62] vs. grade 3 [95% CI 2.74–4.39]). By contrast, non-AYA rated the following items higher on the scale in comparison to AYA patients: fatigue (median grade 5 [95% CI 3.17–4.83] vs. grade 4 [95% CI 3.22–4.42]), aftercare (median grade 5 [95% CI 3.87–5.09] vs. grade 4.5 [95% CI 3.71–4.94]), vaccination (median grade 5 [95% CI 3.92–5.39] vs. grade 4.5 [95% CI 3.93–5.00]) and alternative medicine (median grade 4 [95% CI 3.00–4.56] vs. grade 3.5 [95% CI 2.87–4.34]). However, there were no significant differences between the two patient groups (see Fig. 1). Patients responding within three years after transplantation cited higher informational needs regarding nutrition (median value 4 [95% CI 3.47–4.49]) compared to patients answering more than three years after HSCT (median value 3 [95% CI 2.39–3.82], *p* = 0.04 (*U* test)).

### Psychosocial informational needs

In terms of psychological issues, informational need regarding body shape/sexuality (median grade 3 [95% CI 3.04–3.98]), coping strategies (median grade 4 [95% CI 3.43–4.33]), relaxation techniques (median grade 3 [95% CI 2.90–3.85]) as well as family, relationship and friends (median grade 3 [95% CI 2.87–3.69]) were all of intermediate importance.

**Fig. 2:**
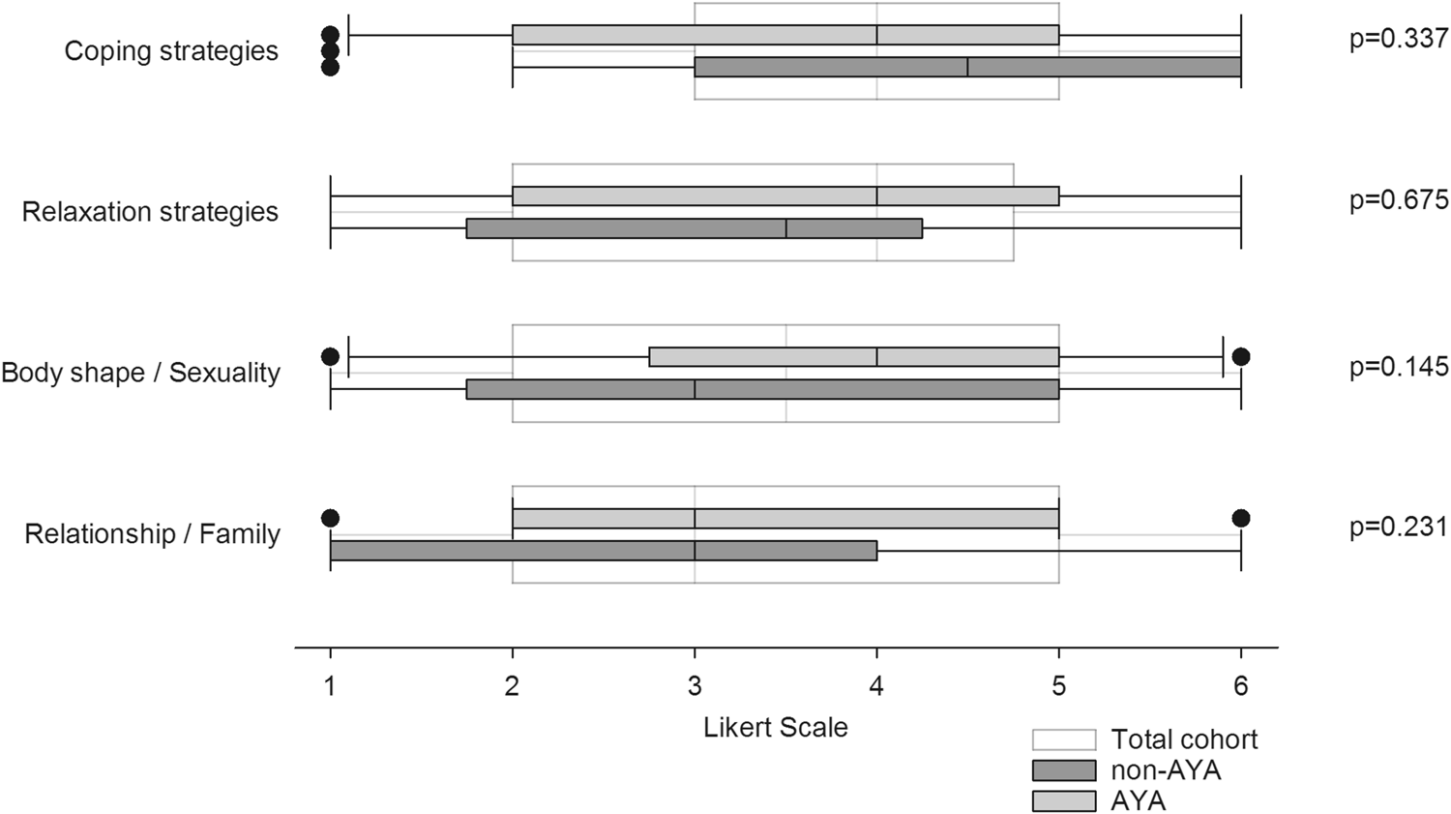
Informational needs regarding psycho-oncologic issues. Boxplots represent median values on a one-to-six graded Likert scale. White boxplots represent the total cohort, grey plots the subgroup of adolescents and young adults (AYA) and dark grey plots represent adults > 39 years of age (non-AYA). Group comparisons were performed using non-parametric *U* test by Mann and Whitney. The referring *p* values are reported

As shown in Fig. 2, AYA in median rated informational needs regarding body shape/sexuality and relaxation strategies one point higher on the Likert scale compared to non-AYA. However, there were no significant differences between the two groups. Time since transplantation (< 3 years vs. > 3 years) had no influence on informational needs concerning psychological issues.

Issues regarding social policy were generally of high interest. Specifically, rehabilitation was given a median score of 5 by both AYA [95% CI 3.40–4.81] and non-AYA patients [95% CI 3.46–4.98]. The topic of premature retirement was also of high interest in both patient groups, rated 5 by the AYA-group [95% CI 3.18–4.54] as well as by the non-AYA patients [95% CI 3.33–5.10]. By contrast, nursing care was of minor interest to both groups with a median rating of 2 (AYA [95% CI 1.78–3.35]; non-AYA [95% CI 1.74–3.12]). Again, time since transplantation (< = 3 years vs. > 3 years) had no influence on the need for information.

### Modality of advice

When asked about the preferred mode of communicating advice, the vast majority of patients (*n* = 49 (82%)) reported a preference for one-to-one conversation in a quiet environment rather than information sessions (*n* = 6 (10%)) or both modalities (*n* = 5 (8%)). When asked about the use of other sources of information, both groups reported use of the internet (*n* = 50 (77%)) or of printed materials (*n* = 40 (62%)), whereas self-help groups were of minor interest. AYAs (*n* = 26; 87%) were most likely to report the use of internet-based advice compared to non-AYA (*n* = 24; 69%, *p* = 0.08 (*χ*^2^ test)). Preferences concerning the mode of communicating advice were not influenced by time since transplantation.

## Discussion

Several previous studies have focused on long-term sequelae of HSCT recipients, especially certain medical survivorship issues such as chronic GvHD or organ dysfunction (Buchsel 2009; Hilgendorf et al. 2015; Tichelli and Rovó 2015). However, HSCT recipients report unmet informational needs on not only medical but also psycho-social topics. The results of the current study can therefore be summarized as follows:

1. Among patients who underwent HSCT medical information is in highest demand, followed by information on social and psychological issues.
2. AYA may require more information on psychological issues compared to non –AYA patients after allogeneic HSCT.
3. AYA were more interested in information regarding nutrition and body shape/sexuality, whereas non-AYA cited higher demand for information on vaccination, aftercare, fatigue and coping strategies.
4. One-to-one conversation is the preferred mode of communicating advice for both AYA and non-AYA patients. Internet-based information platforms were of higher interest to AYA patients than non-AYA patients.
5. Nutrition information is an important topic particularly within the first three years after HSCT.

Although HSCT may cure the underlying disease, successful treatment often cannot be equated with a full restoration of health (Wingard 2002). HSCT recipients may suffer from persistent health issues, including fatigue, sexual dysfunction, chronic GvHD, pain, and the inability to return to work. With special focus on AYA patients, Cooke et al. (Cooke et al. 2011; Persoon et al. 2013) reported physical, psychosocial, existential and other issues emerging after HSCT. The AYA HOPE study ascertained that between 25 and 50% of AYAs had unmet informational needs regarding fitness, opportunities to meet other AYA survivors, nutrition and diet, financial support, fertility and the risk of family members getting cancer (Keegan et al. 2012). Recently, Mathanda et al. (2020) evaluated quality-of-life (QoL) trajectories in AYAs using the FACT-BMT questionnaire; they found that, unlike older patients, the AYAs’ QoL values before and after HSCT were comparable. In line with the current study, Lahaye et al. (2017) describe the presence of ongoing physical and psychosocial effects as consequences of the past illness and treatments in AYA survivors after HSCT. Our study confirms these findings and further specifies the relative importance of the medical, psychological and social issues of most interest to HSCT recipients, especially to the AYA cohort.

Because long-term survivors of HSCT have an increased risk for metabolic syndrome as well as cardiovascular morbidity (DeFilipp et al. 2016), it is important to improve modifiable risk factors. A moderate or a bad nutritional intake after cancer treatment was reported by 73.9% and 21.7% of AYA participants, respectively. Intensive nutrition counseling appears to improve nutritional behavior (Quidde et al. 2016). In our study, AYA emphasized a higher level of informational need on the topic of nutrition compared to older patients, which is consistent with previous reports of cancer survivors (Beckjord et al. 2008; Zebrack 2008).

In the current study, AYA patients expressed a higher demand for information regarding body shape and sexuality issues. In addition, AYA suffering from hematologic malignancies emphasized sexual functioning and intimacy as one of the relevant issues before and after treatment (Husson et al. 2018). As patients are likely to believe that sexual dysfunction and treatment-related damage on reproductive organs cannot not be treated (Bober and Varela 2012), information on these topics should be targeted especially to AYA patients.

Fatigue affects various aspects of life such as physical activity, concentration or psychosocial adjustment (Hilarius et al. 2011). With a special focus on AYA, cancer-related fatigue has been found to be highly prevalent and to occur more often in AYA than in non-AYA cancer patients (Nowe et al. 2017). The reported high demand for information regarding fatigue in the current study is not surprising, as cancer-related fatigue represents one of the most common symptoms after HSCT (Bevans et al. 2008). However, non-AYA patients indicated a higher demand for information regarding fatigue than did AYA. This may be explained by a higher maximum and adjusted activity scores of the human activity profile scale (HAP) of AYA compared to non-AYA after HSCT (Pulewka et al. 2017) and the known positive impact of exercise on fatigue and quality of life. Rehabilitation strategies including physical activity, psychoeducation, cognitive therapy and peer support are promising approaches to reducing fatigue and distress (van Weert et al. 2005; Thorsen et al. 2011). Therefore, post-transplant information should include these topics.

Our study also found a high demand for information on current recommendations for vaccination after HSCT. This is important, since vaccine-preventable infections are a significant cause of morbidity, re-hospitalization and mortality after HSCT (Tsigrelis and Ljungman 2016). Since AYA more often describe themselves as less conscientious in comparison to non-AYA (Pulewka et al. 2017), and therefore may not adhere to vaccination standards, vaccination after HSCT should be discussed carefully with them.

Issues regarding social policy were generally of high interest in this study. Questions regarding rehabilitation opportunities or premature retirement are crucial for HSCT recipients, because it may enable the patient to make plans for their future. HSCT survivors often suffer from a diversity of handicaps, impairments and disabilities (Steinberg et al. 2015). Long-term survivors' engagement in paid work is still influenced even many years after treatment, which confirms the need to include vocational assistance in post-SCT rehabilitation (Winterling et al. 2018). Therefore, structured rehabilitation programs are required to lessen the effects of negative sequelae of HSCT.

Social policy issues were of high interest among our study participants. In this respect, the YOUNG CANCER PORTAL (https://www.junges-krebsportal.de/) facilitates contact with experts in social medicine throughout Germany and access to necessary information and advice for AYA with cancer. The online counseling portal covers social law issues but also various other topics, such as changes in hormone balance, immunodeficiency and integrative cancer medicine.

Most of the patients in our study preferred a one-to-one communication process rather than self-help groups. This result highlights the need for personal conversation within this special patient cohort and is in line with an Australian survey reporting that the majority of patients preferred long time follow up with their transplant physician (Dyer et al. 2016). At our institution we offer a survivorship care plan and lifelong surveillance following allogeneic HSCT.

Asked for the mode of information, especially AYA patients reported to use the internet (Perales et al. 2016; Domínguez and Sapiña 2017). This finding can be supported by the present study. Other studies described benefit of using internet interventions in patients with chronic disease (Murray et al. 2005). With special focus on patients undergoing HSCT, specifically designed websites are described including information about the transplant procedure, supportive and survivorship care (Syrjala et al. 2011; Horne et al. 2016). However, although AYA might use the internet, the quality of some content was questioned and did not meet the AYAs needs and expectations (Mooney et al. 2017). Webinars or other supervised online forums may be beneficial to address the desired information.

In addition to its strengths, the limitations of this study are the single center and cross-sectional design resulting in a relatively low number of participants as well as a lack of data on how needs may evolve over time. Moreover, we used a non-validated questionnaire for the survey. Thus, the results of the study may be interpreted in terms of particular considerations rather than generalizations. Nevertheless, our study truly reflects the everyday life of this special patient cohort.

In conclusion, despite well-established aftercare projects with a focus on sequelae after HSCT, the majority of patients describe unmet informational needs in medical, psycho-oncological and social-care topics. AYA and non-AYA patients have different informational needs after HSCT. To gain further insight into this matter, a prospective study has been initiated in which data is collected at four specified time points after HSCT.
